# Using sibship reconstructions to understand the relationship between larval habitat productivity and oviposition behaviour in Kenyan *Anopheles arabiensis*

**DOI:** 10.1186/s12936-019-2917-5

**Published:** 2019-08-23

**Authors:** Joel O. Odero, Ulrike Fillinger, Emily J. Rippon, Daniel K. Masiga, David Weetman

**Affiliations:** 1 Human Health Theme, International Centre of Insect Physiology and Ecology, Nairobi, Kenya; 2 Vector Biology Department, Liverpool School of Tropical Medicine, Liverpool, UK

**Keywords:** Microsatellites, *Anopheles arabiensis*, Larval control, Skip-oviposition, Auto-dissemination, Sibship-reconstruction

## Abstract

**Background:**

Strategies for combatting residual malaria by targeting vectors outdoors are gaining importance as the limitations of primary indoor interventions are reached. Strategies to target ovipositing females or her offspring are broadly applicable because all mosquitoes require aquatic habitats for immature development irrespective of their biting or resting preferences. Oviposition site selection by gravid females is frequently studied by counting early instar larvae in habitats; an approach which is valid only if the number of larvae correlates with the number of females laying eggs. This hypothesis was tested against the alternative, that a higher abundance of larvae results from improved survival of a similar or fewer number of families.

**Methods:**

In a controlled experiment, 20 outdoor artificial ponds were left uncovered for 4 days to allow oviposition by wild mosquitoes, then covered with netting and first and second instar larvae sampled daily. Natural *Anopheles* habitats of two different types were also identified, and all visible larvae sampled. All larvae were identified to species, and most samples of the predominant species, *Anopheles arabiensis*, were genotyped using microsatellites for sibling group reconstructions using two contrasting softwares, BAPS and COLONY.

**Results:**

In the ponds, the number of families reconstructed by each software significantly predicted larval abundance (BAPS R^2^ = 0.318, p = 0.01; COLONY R^2^ = 0.476, p = 0.001), and suggested that around 50% of females spread larvae across multiple ponds (skip oviposition). From natural habitats, the mean family size again predicted larval abundance using BAPS (R^2^ = 0.829, p = 0.017) though not using COLONY (R^2^ = 0.218, p = 0.68), but both softwares once more suggested high rates of skip oviposition (in excess of 50%).

**Conclusion:**

This study shows that, whether in closely-located artificial habitats or natural breeding sites, higher early instar larval densities result from more females laying eggs in these sites. These results provide empirical support for use of early instar larval abundance as an index for oviposition site preference. Furthermore, the sharing of habitats by multiple females and the high skip-oviposition rate in *An. arabiensis* suggest that larviciding by auto-dissemination of insecticide may be successful.

## Background

Concerted efforts towards malaria control and elimination have led to a global decline in malaria cases by about 40% between 2000 and 2015 [1]. Almost 80% of this reduction is attributed to vector control by widespread distribution of insecticide-treated nets (ITNs) and indoor residual spray (IRS) [1]. Despite these successes, malaria reduction in the World Health Organization (WHO) African region has stalled [2], which may in part reflect limitations of ITNs and IRS. These limitations include plasticity in behaviours including early biting and outdoor resting, and feeding on animals allowing adult malaria vectors to avoid exposure to insecticides [3, 4], widespread insecticide resistance [5, 6], and high operational costs of IRS in particular which limit coverage [2]. Implementation of supplementary vector control tools is required to further reduce malaria transmission in a trajectory toward elimination [7, 8].

Larval source management (LSM) is a tool for further development because all mosquitoes need to lay eggs in an aquatic habitat irrespective of their biting or resting preferences [9]. However, the uptake of this intervention is impeded by the management effort required [10] and the lack of knowledge of aquatic habitats that are the most preferred for egg laying, which would allow a more spatially-targeted approach. The oviposition behaviour of *Anopheles* mosquitoes, and specifically the preference of particular aquatic habitats for egg-laying, has been studied in order to better target LSM and to develop novel attract and kill strategies for vector control [11, 12]. Habitat preferences are frequently inferred from the abundance of early instar larvae in a habitat [13]. This relies on the assumption that higher early instar larval density results from a greater number of gravid females selecting the site for oviposition [11, 12]. However, to date only indirect tests of this assumption exists [14]. Furthermore, whilst there is strong indication from cage experiments that species of the *Anopheles gambiae* complex frequently distribute their eggs in more than one egg-laying sites (skip-oviposition) [15], this behaviour has not yet been widely accepted in *Anopheles* due to the few studies providing supporting evidence [14].

An LSM tactic currently being explored is the auto-dissemination of insect growth regulators (IGRs) where adult mosquitoes naturally transfer the IGR from resting to breeding sites [16, 17]. Large numbers of females visiting multiple breeding sites would aid transfer of IGR among habitats to achieve biologically-relevant mortality of the immature stages. However, skip oviposition has been widely accepted to occur habitually in *Aedes* mosquitos unlike in *Anopheles* [18–20]. In typical habitats of western Kenya early instar densities vary from 160/m^2^ in puddles to between 0.3 and 10/m^2^ in open drainages, cultivated swamps, and river fringes [13, 21]. *Anopheles* females can lay up to 200 eggs [15], and it is unclear whether the typically-low densities of early instars are a result of few gravid females visiting most habitats or of high mortality of the eggs or larvae from many females.

Here genotype-based family reconstructions methods were used to enumerate the number of female *Anopheles arabiensis* depositing eggs in single and multiple habitats in relation to overall larval densities. It was hypothesized that relative high number of early instar *An. arabiensis* larvae in a habitat is an indicator of high number of females laying eggs in a site; therefore, differences in early instar abundance across similar habitat types of comparable size correlate with the number of females laying eggs.

## Methods

### Collection and rearing of *Anopheles arabiensis* mosquito families for relatedness testing

Wild blood-fed *Anopheles* females were collected in May 2015 by aspiration from a cattle-baited trap [22] set up in Kirindo village, Mbita sub-county (0°26′38.46″S 34°15′36.95″E), western Kenya. *Anopheles gambiae* sensu lato (s.l.) females were identified morphologically, and other mosquito species discarded. Individual females were held in paper cups covered with a fine net. Moist cotton wool covered with filter paper was provided at the base for egg laying. Food was provided ad libitum by a disc of cotton wool soaked with 10% glucose solution on top of the cup. After egg laying, single legs from individual females were genotyped to identify species [23] to ensure that only *An. arabiensis* families were reared. *Anopheles arabiensis* eggs from each mother were raised separately in trays and the mothers preserved individually at − 80 °C in Eppendorf tubes containing absolute ethanol. After hatching, larvae were fed twice daily on TetraMin fish food (Tetra, Germany) and pupal stages from each family were separated into females and males prior to emergence to prevent mating. Following emergence, single virgin females and three males from different families were held in 15 × 15 cm mosquito cages for 48 h to copulate. Females were then offered two blood meals and held in paper cups to lay eggs, with resulting offspring reared to early instar stage. All the early instar larvae offspring were preserved at − 80 °C in Eppendorf tubes (20 larvae/tube) containing absolute ethanol for subsequent DNA analyses.

### Standardized semi-field evaluation

Semi-field experiments were conducted at the International Centre for Insect Physiology and Ecology (ICIPE), Mbita, western Kenya, between May and June 2016. Twenty artificial ponds were created in a field on campus using plastic tubs (40 cm diameter, 20 cm deep) fully buried in the ground and filled with 50 l of untreated water originating from Lake Victoria to mimic typical *Anopheles* mosquito breeding habitats. The ponds were set up in a 4 × 5 pond grid with neighboring ponds in each gridline 4 m apart. Ponds were left open for 4 days to allow for natural colonization by egg-laying wild female mosquitoes and then covered with netting on day 5 to prevent further egg laying. All visible larvae were sampled exhaustively daily for a further 6 days to allow time for most of the eggs to hatch. Larvae from each habitat were preserved at − 80 °C in 15 ml Falcon tubes containing absolute ethanol for further analysis.

### Field survey of natural aquatic mosquito larval habitats

Field surveys and larval sampling from natural aquatic habitats was conducted between March and May 2017 within a rice irrigation scheme (00°28.473′S 034°32.786′E) (see Additional file 1). Two common mosquito larval habitat categories in the area—puddles and drainage ditches—were identified. Six puddles and five ditches of a perimeter size between 0.64 and 11 m^2^ were the selected, and exhaustively sampled using larval sweep nets. The estimated pairwise distance between the habitats ranged from 2 m between drainage 2 (D2) and puddle 7 (P7) to 1264 m between puddle 1 (P1) and puddle 8 (P8) *Anopheles* larvae were separated from the collections morphologically and preserved at − 80 °C in 15 ml Falcon tubes containing absolute ethanol.

### DNA extractions and species identification

Genomic DNA was extracted from: (i) the two *An. arabiensis* families (Family A—adults n = 4, larval offspring n = 46; Family B—adults n = 4, larval offspring n = 36) collected from cattle-baited traps; (ii) the *An. gambiae* s.l. larvae collected from the artificial pond habitats (n = 466); and (iii) the natural field habitats (n = 702). Samples were extracted using the Nexttec DNA isolation kit (Biotechnologie, GmbH) following manufactures instructions. Following PCR-based species identification [23], but prior to further analysis, *An. arabiensis* samples from different artificial ponds and natural habitats were randomized on DNA extraction plates to mitigate any bias from plate batch effects.

### Microsatellite genotyping

Fifteen *An. arabiensis* microsatellite DNA markers located on chromosomes 2 and 3 were used in this study (Table 1). Markers were allocated into four multiplexes based on compatible primer annealing temperatures and non-overlapping expected allele size ranges using Multiplex Manager software [24]. Forward primers were fluorescently labelled with either NED, VIC, 6-FAM, or PET dyes. Amplification was carried out using the Qiagen Type-it microsatellite PCR kit with each reaction consisting of 6.25 μl Type-it master mix, 1.25 μl primer mix, 3 μl water, and 2 μl DNA template. The thermocycling conditions were as follows: initial activation at 95 °C for 5 min, 30cycles of denaturation at 95 °C for 30 s, annealing at 57–63 °C for 90 s, extension at 72 °C for 30 s with a final extension at 60 °C for 30 min. The PCR fragments were separated on an ABI 3730XL (Applied Biosystem) sequence analyser using the GeneScan™–500LIZ™ size standard. The allele sizes were scored using GeneMarker software v.2.6.7 (SoftGenetics) with each allele size score checked manually.

**Table 1:** Details of microsatellite primer sequences used for the genotyping

Plex	Marker (label)	Concentration (μM)	Chromosome	Genetic location	Forward sequence (5′–3′)	Reverse sequence (5′–3′)
1	CDC675-FAM [33]	1.75	2L	24C	TCAAACTCGAACTCCTCAAC	TTTCCGTCGATAGTTTTCTG
	CDC22-FAM [33]	1.75	2L	22D	GGGCAAAGAGAAAGCAA	AGCTGTGTGGCAGGTTT
	CDC46-NED [33]	2.5	3L	45C	GTGGTTGACCGATTTGTAAG	ATTTATTCACTCGCCAAGAA
2	CDC18-FAM [33]	1.9	2R	18C	CAGGAAGCGATGTGAAAGT	GGAGTGTTGTCGTTCATCTT
	CDC28C-FAM [33]	2.3	2L	28C	TGTGCCGGTTGAGAGAGA	GGGCGAGAACATTAACAA
	Ag2:79-NED [34]	1.9	2R	11B	CGGGTAGCGCTAGAAGTATG	AGAGAAATGTGCCGAAGGGG
	2RiS5-PET [35]	1.9	2R	12C	TTCTCGAAAGACTGCTGCTG	ATTGGATCGAAAACGGTCTG
3	CDC40B-FAM [33]	1.9	3L	40B	ATGCATGCAAATCGGTAT	TATCGAGGCAAATCGGTA
	CDC44-VIC [33]	2.3	3L	44B	ATGCATGCAAATCGGTAT	TATCGAGGCAAATCGGTA
	CDC32-NED [33]	1.9	3R	32A	GTTTGCTTGCTTGTTGTTGT	GTGCTCAACGCCTACAAAT
	CDC34-PET [33]	1.9	3R	34B	AAAACTTTTCCCTCCCATTC	AAGTGCAGCAATTGACGAG
4	Ag3:128-FAM [34]	1.9	3R	29C	CGGGACGGCTAGATAAAGCG	CCGGGCGACATAACCCACCC
	Ag2:143-VIC [34]	1.5	2L	25D	CGTACGAGTGAGTGAGTTGG	CAAAAATAGCATCACGGCCG
	Ag2:46-NED [34]	2.7	2R	7A	CGCCCATAGACAACGAAAGG	TGTACAGCTGCAGAACGAGC
	Ag3:249-NED [34]	1.9	3R	30B	ATGTTCCGCACTTCCGACAC	GCGAGCTACAACAATGGAGC

The markers were grouped into four PCR multiplexes

### Family group assignment

Genotyping errors possibly due to null alleles, large allele dropouts and mis-scoring of stutter peaks were identified using MICRO-CHECKER v.2.2.3 [25].

The capacity of the markers to determine sibships within known pedigrees was first tested using the two *An. arabiensis* families, A and B, with known parents. Family reconstruction was performed using two programs, COLONY [26] and BAPS 6 [27], that differ fundamentally in their methodologies and thus provide independent analyses. COLONY implements a likelihood algorithm in which individuals are simultaneously inferred into groups with the aim of finding the most likely grouping. In COLONY a full-likelihood (FL) analysis algorithm was used without a sibship prior and using a pool of allele frequencies from the natural habitats as a reference population. BAPS implement a Bayesian clustering algorithm treating both sample allele frequencies and the number of larval family groups as random variables when inferring clusters. In BAPS an individual clustering analysis was performed with the number of possible clusters ranging from 5 to 100.

### Statistical analysis

**Fig. 1:**
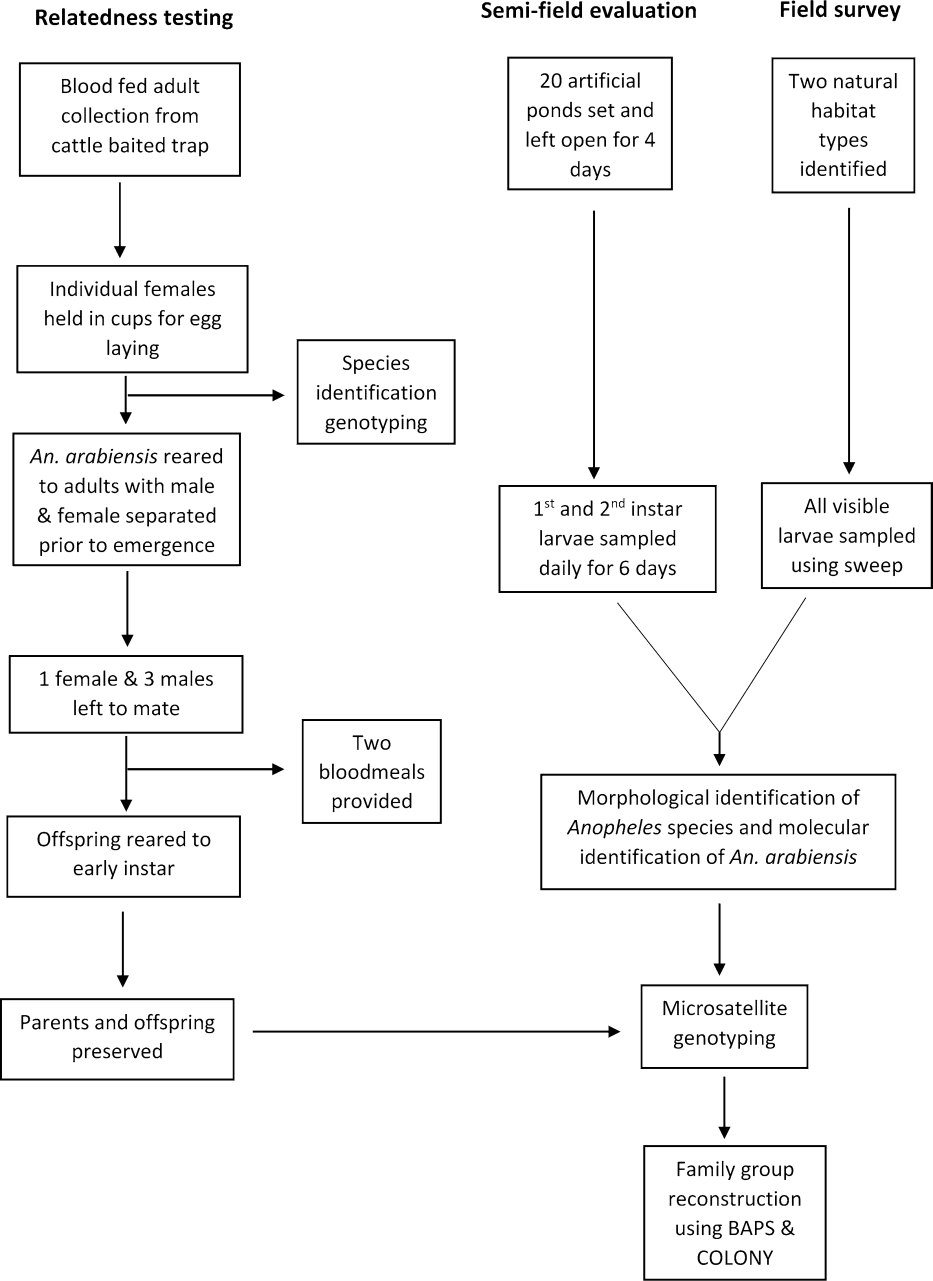
Flowchart of the experimental design and analysis methods used in the study

A Kolmogorov–Smirnov test was used to check for normality of the distributions for numbers of larvae and reconstructed families, followed by a Grubbs test to check for outliers in the data, which were removed if detected. Pearson correlation and linear regression were used to test the relationship between the number of families and larval abundance. A partial Mantel test was used to test whether large families were more likely to be split across multiple habitats and if split families were more likely to occur in closer habitats in the software ZT [28]. For this test, a dummy (1/0) variable indicated whether habitats were direct neighbours or not, whilst controlling for sample size (as the minimum sample size per pair of habitats). The different experimental designs and analysis methods used in this study have been summarized in Fig. 1.

## Results

### Marker testing using known sibship

The capacity of 15 microsatellite markers, developed for *An. arabiensis*, to determine relatedness within known pedigrees was tested using two families of known full-siblings (A: n = 46 offspring; B: n = 36 offspring). The genotype from the mothers in Family B were inconclusive because of lack of informativeness of the loci, with some genotypes missing and therefore, only genotypes from family A were used for determination of parent–offspring Mendelian segregation but both families were used for sibship analysis. Twelve of the 15 markers yielded segregation patterns between the parents and amongst full-sibs in family A or amongst full sibs in family B consistent with Mendelian inheritance (see Additional file 2), and these markers were retained for subsequent analyses. Clustering analysis using either COLONY or BAPS provided entirely concordant results and correctly identified a single cluster of siblings within each family with no cross-family assignment.

### Relatedness among the standardized artificial ponds

Eighteen of the twenty artificial ponds were colonized, with a total of 466 *Anopheles* larvae collected. The total number of larvae collected from each pond ranged from 4 to 62 (median = 16.5, inter-quartile range = 31) (Table 2). From the species identification genotyping, 96.8% (n = 451) of the larvae were identified as *An. arabiensis* and were subsequently genotyped using microsatellites, 1.9% (n = 9) *An. gambiae* sensu stricto (s.s.), with 1.3% (n = 6) failed reactions.

**Table 2:** The number of mosquito families estimated using BAPS and COLONY in the artificial ponds

POND	N	n	BAPS	COLONY
A	42	37	6	13
C	5	4	1	1
D	40	40	5	6
E	8	6	5	5
F	10	10	3	9
G	17	14	5	6
H	33	26	2	7
I	5	4	3	3
J	59	44	6	9
K	41	36	5	11
L	52	23	3	4
M	62	60	6	10
N	4	4	2	2
O	39	34	2	5
P	13	9	2	4
Q	16	16	4	5
R	10	8	5	6
T	10	10	3	5

N is the total number of *An. arabiensis* larvae from each pond, and n is the number successfully genotyped

**Fig. 2:**
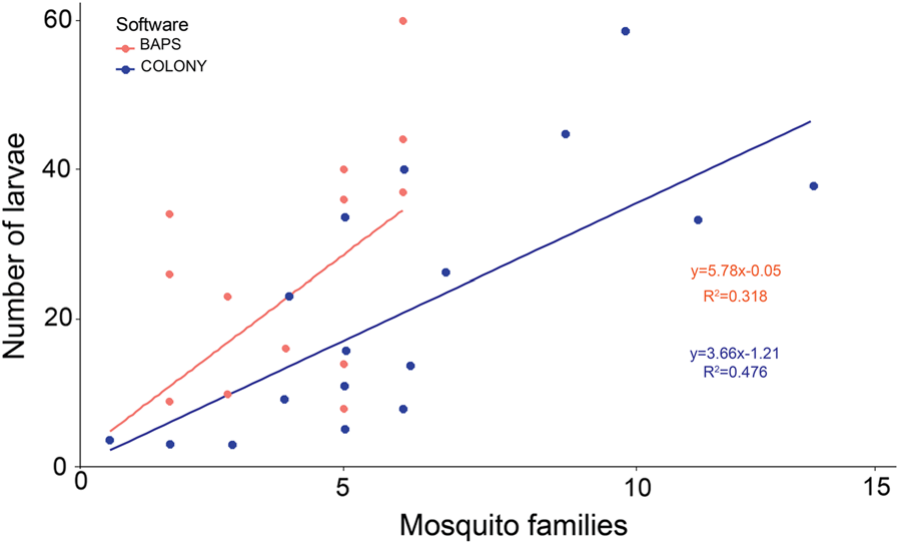
Relationship between mosquito family clusters inferred using COLONY and BAPS with the total number of larvae from each artificial pond habitat

COLONY estimated on average more females per pond compared to BAPS (Table 2), but the results were well correlated across families (r = 0.715, p < 0.001). Overall, BAPS estimated a total of 28 gravid females to have laid eggs across the 18 ponds while the COLONY analysis suggested 70 gravid females. Despite the difference in the total number of families estimated, 95% of the pairwise assignments of individuals as siblings in COLONY were also identified by BAPS, but the latter then appears to reconstruct many additional family pairings within its much larger clusters. In both clustering methods, the total number of larvae in each pond significantly predicted the number of families reconstructed (BAPS R^2^ = 0.318, p = 0.01; COLONY R^2^ = 0.476, p < 0.001) (Fig. 2). This corroborated the hypothesis that a relatively higher number of larvae in a pond is indicative of a higher number of females laying eggs in the pond as compared to a pond with a lower number of larvae in close vicinity.

**Fig. 3:**
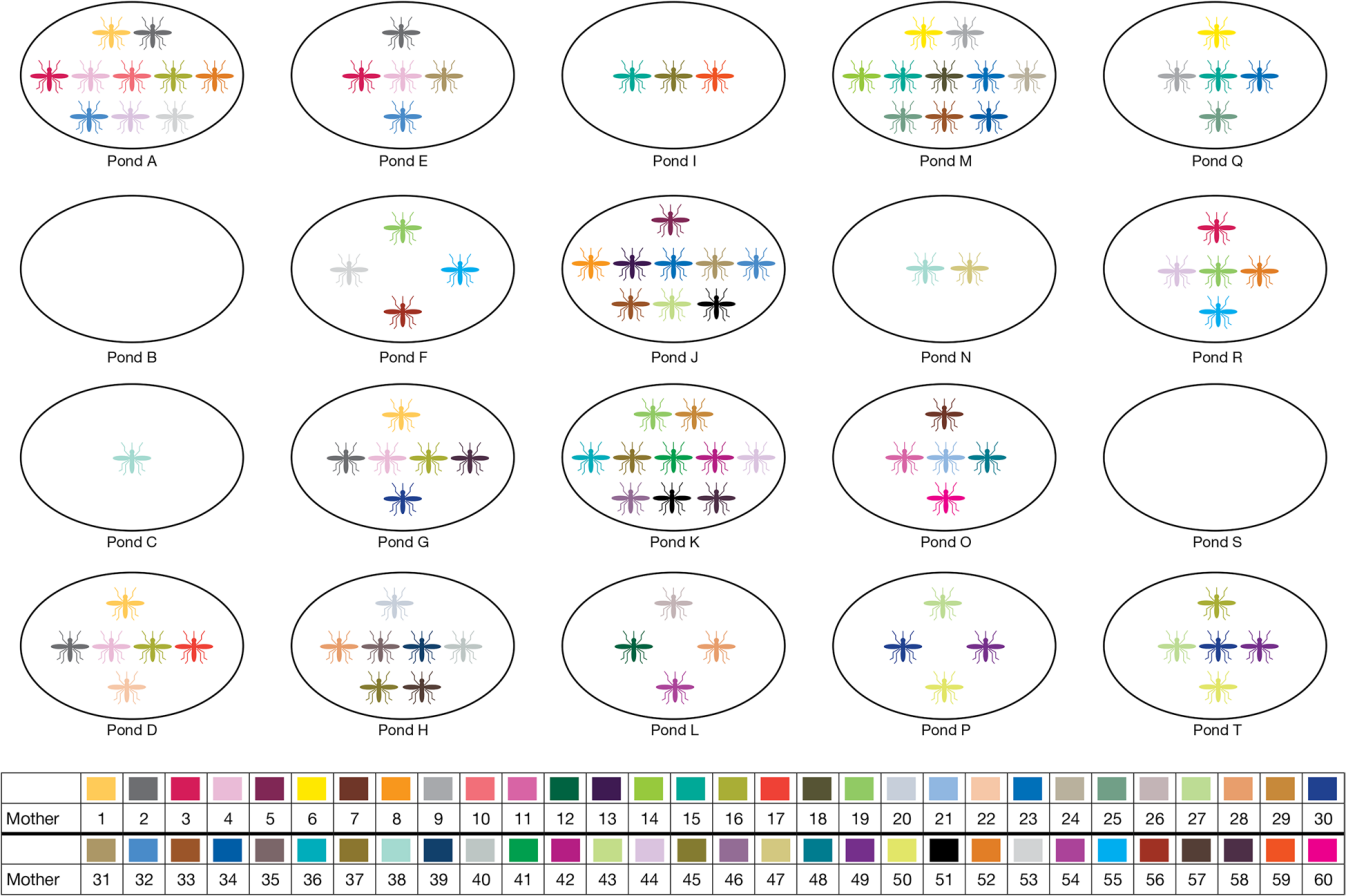
Pond setup and mosquito family distribution across the 18 ponds as inferred using BAPS. The colours represent families from a single mother

**Fig. 4:**
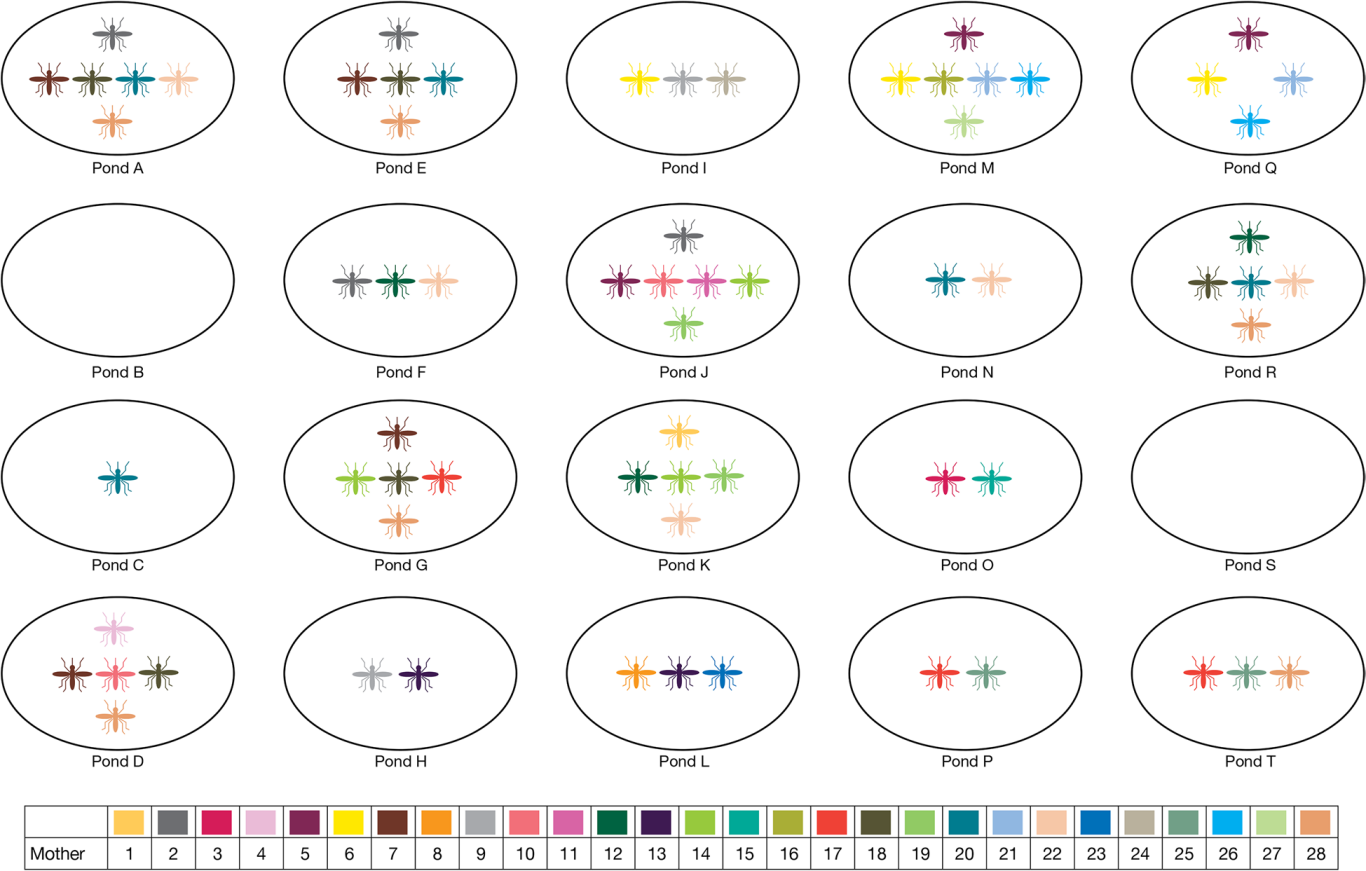
Pond setup and mosquito family distribution across the 18 ponds as inferred using COLONY. The colours represent families from a single mother with only families with two or more offspring visualized (n = 60)

Average numbers of larvae per family were quite low but very variable (median, IQR-BAPS 4.3, 4.9; COLONY 3.0, 2.9) suggesting high mortality of unsampled first instar larvae or eggs. Overall, BAPS estimated that 57% (95% CI 39, 75) females to have deposited larvae in multiple ponds and COLONY 43% (95% CI 30, 56), suggesting a high frequency of skip-oviposition by the mothers (Figs. 3, 4). Given the high skip-oviposition estimates, two linked questions were asked: are larger families more likely to be split across ponds, and are split families more likely to occur in closer ponds? There was a significant correlation between the number of larvae from each mosquito family and the number of ponds in which they were found using either estimator (BAPS r = 0.46, p = 0.015; COLONY r = 0.35, p = 0.003). Dependence of family sharing on pairwise proximity of pond pairs, while controlling for the minimum sample size per pair of ponds, was also evaluated in a partial Mantel test. There was a non-significant result for BAPS (r = 0.107 p = 0.11) and a marginally significant correlation for COLONY (r = 0.15 p = 0.04). This suggests that over the small spatial scale examined in the experimental pond design (Figs. 3, 4), larger families were more likely to be split but the preference of females to lay their eggs in physically closer habitats was weak.

### Relatedness in natural habitats

A total of 702 larvae were sampled from across 11 natural habitats with the total number of larvae in each habitat ranging from 28 to 109 (median = 41, IQR = 41) (Table 3); 79% (n = 556) were identified as *An. arabiensis,* which were genotyped using microsatellites, 9.7% (n = 68) *An. gambiae* s.s., and 11.1% (n = 74) were failed reactions, some of which may have been non-*An. gambiae* s.l. Owing to much greater variability in genotyping success in the natural habitat samples, mean family size (number of larvae genotyped divided by number of families) was used as a metric for analysis rather than the number of mosquito families as in the ponds. Larval counts from habitat 8 was found to be an outlier compared to the other 11 natural habitats (p < 0.05) and was excluded from the analysis. After this exclusion all the larval counts from all habitats were normally distributed, Kolmogorov–Smirnov test p > 0.05.

**Table 3:** The number of mosquito families estimated using BAPS and COLONY in the natural habitats

Habitat	Habitat size (M)	N	n	BAPS	COLONY	Mean family size	
						BAPS	COLONY
P1	0.6	28	18	9	13	2.0	1.4
P5	0.3	36	22	13	20	1.7	1.1
P6	1.4	37	25	14	19	1.8	1.3
P7	1.3	66	10	6	9	1.7	1.1
P8	6.9	191	97	21	75	4.6	1.3
P9	2.6	55	51	17	37	3.0	1.4
D1	11.0	33	30	16	25	1.9	1.2
D2	5.8	109	60	19	42	3.2	1.4
D3	1.9	41	15	9	12	1.7	1.3
D9	3.7	33	24	14	18	1.7	1.3

N is the total number of *An. arabiensis* larvae from each habitat, and n is the number successfully genotyped. In the habitat column, prefix P is puddle habitats while D is drainage ditches

**Fig. 5:**
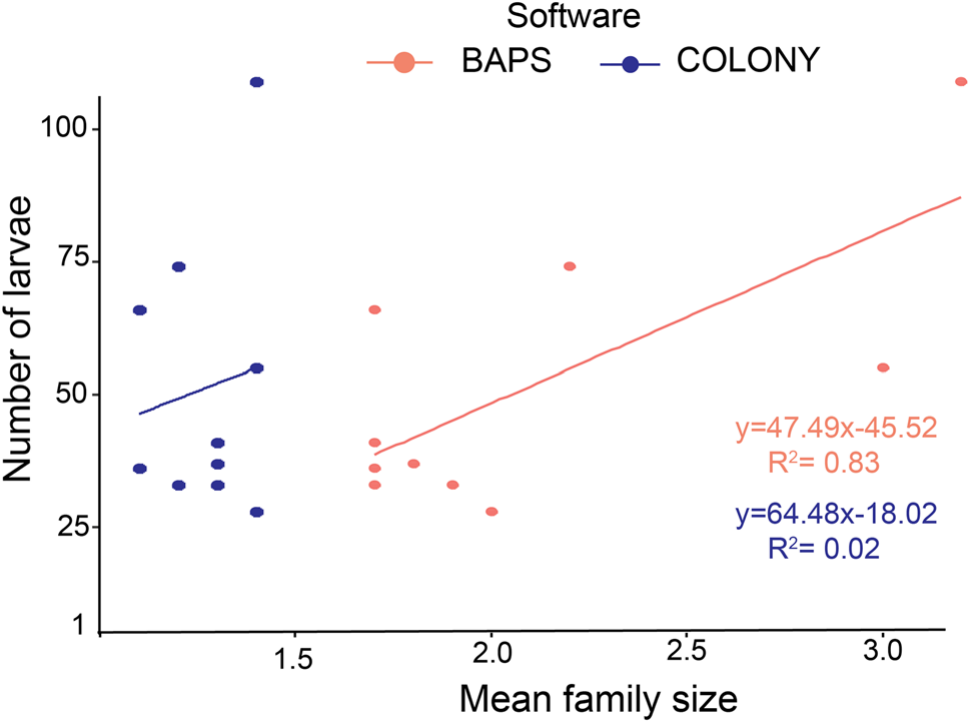
A regression curve comparing relationship between mosquito families inferred using COLONY and BAPS with the total number of larvae from natural habitats

Once again COLONY estimated many more families (N = 171) compared to BAPS (N = 41) but the results for mean family sizes estimated using each method were marginally correlated (r = 0.634, p = 0.049). Despite this correlation, only 56% of the pairwise sibship assignments in COLONY were also detected by BAPS suggesting a more moderate agreement between the two software than in the pond experiment. Mean family sizes from the BAPS estimation predicted larval abundance but those estimated by COLONY did not (BAPS R^2^ = 0.829, p = 0.017; COLONY R^2^ = 0.218, p = 0.68) (Fig. 5). Similarly, only results from the BAPS analysis supported the hypothesis that a higher number of larvae in one habitat compared to another at the same time is indicative of a higher number of females choosing to lay eggs. Nevertheless, both estimators suggested once again a high frequency of skip-oviposition (BAPS = 73%; 95% CI 59, 87; COLONY = 60%; 95% CI 53, 67.

Analysis to answer the same two linked questions as before, found that the mean family size was significantly positively correlated with the number of habitats among which they were distributed using BAPS (r = 0.902, p < 0.001) or COLONY (r = 0.451, p < 0.001). The tests of whether family sharing was dependent on proximity of habitat pairs were both non-significant (BAPS partial Mantel test r = 0.104, p = 0.22; COLONY r = − 0.027, p = 0.43).

## Discussion

Understanding mosquito oviposition behaviour is important to inform the development and implementation of breeding habitat-targeted mosquito control strategies. However, direct observational studies of oviposition behaviour are challenging due to its nocturnal occurrence. As such, limited evidence exists to support phenomena such as habitat preference, variations in larval densities across breeding habitats and frequency of skip oviposition by *An. gambiae* complex females.

In this study, genetic clustering (BAPS) and sibship reconstruction (COLONY) methods were applied to enumerate the number of female *An. arabiensis* depositing eggs in single and multiple habitats in relation to overall larval densities. The findings show that relative high number of early instar larvae in a breeding habitat is indicative of an increased number of ovipositing females. This supports the hypothesis that early instar larval abundance of *An. arabiensis* is an indicator of the number of females laying eggs in a habitat and can hence be used to investigate oviposition preferences. This finding is consistent with a study that previously found a positive correlation between the number of mosquito families and number of larvae in a breeding site [29]. The family sizes detected were generally low, concordant with previous studies [14, 29], and the discrepancy between potential an actual family sizes maybe attributable to low egg hatchability and first instar survival rates [30].

This study also found that *An. arabiensis* females often display skip oviposition, distributing their eggs in multiple habitats. On average, more than half of the families investigated from the experimental and natural habitat surveys resulted from skip-oviposition. The ponds and natural habitats were in a small spatial scale, which could have contributed to the high skip oviposition rates. Since gravid *Anopheles* females tend to hover over habitats before deciding to lay eggs, it was not surprising that skip-oviposition occurred over the entire range. This finding is similar to a study on *An. gambiae* s.s., which found that 57% of females had skip-oviposited in habitats [14]. In contrast, a much lower skip-oviposition frequency of 26% was observed in two-choice cage assays on *An. gambiae* s.s. oviposition in the laboratory [15]. The low frequencies observed in this cage assay could have been due to the individual mosquitoes responding to a similar oviposition substrate reducing preference to skip oviposit. There was also a relationship between mosquito family size and the number of habitats in which they were distributed in both the artificial ponds and natural habitats implying that females that laid more eggs were more likely to exhibit skip oviposition.

Frequent skip-oviposition behaviour could contribute positively to the success of *An. arabiensis* mosquito control methods using auto-dissemination of insect growth regulators (IGRs) where skipping adult females naturally transfer the IGR from resting to breeding sites. *Anopheles arabiensis* have been shown to experimentally transfer pyriproxyfen from their resting sites to aquatic habitats leading to significantly reduced larval emergence [16, 17]. Our results suggest that skip oviposition behaviour should also be considered as an important factor in such studies and in the ecology of these vectors.

The number of families estimated using BAPS were consistently much lower than COLONY both in the artificial ponds and natural habitats. This could been in part due to a tendency of COLONY to over-split large families as previously noted in simulation studies [31, 32]. Also, whilst both estimators supported the relationship that high larval density in a breeding habitat is indicative of an increased number of ovipositing females in the ponds, only BAPS did so in the natural habitats. However, despite contrasting family sizes between the two software, individual pairwise sib-ship assignments made by both agreed strongly in the ponds, although the correspondence was more moderate in the natural habitats. This may reflect a problem with ‘self-referencing’ in COLONY, whereby in the natural habitats the same genotype set was used as those assigned, rather than a wider set fully representing genetic diversity of the population. A similar observation was made in the two families of known full-siblings and in the pond samples when ‘self-referencing’ with COLONY.

## Conclusion

The current study demonstrates the potential use of *An. arabiensis* larval abundance in breeding habitats as an indicator of oviposition site preference and provides empirical evidence of frequent skip-oviposition behaviour, which should be considered when studying their ecology and applying larval control methods. These findings support previous findings for *An. gambiae* s.s., but at present little is known about oviposition behavior in the other major East African malaria vector *Anopheles funestus*, and studies are now warranted.

## Supplementary information

**Additional file 1.** Schematic view of larval sampling sites at Oluch-Kimira rice irrigation scheme in Homa-Bay county, western Kenya. In the site labels, prefix P is puddle while D is drainage ditch.

**Additional file 2.** Mendelian test for allele inheritance for parents and offspring in family A. The markers with p values in bold indicate consistency with Mendelian allele segregation rule. N/A represent markers that did not amplify, and n/a represent homozygous alleles whose p values could not be calculated.

## Data Availability

The datasets used and/or analysed during the current study are available from the corresponding author on reasonable request.

## Abbreviations

LSM: larval source management
IGRs: insect growth regulators
CBT: cattle baited traps
ITN: insecticide-treated net
IRS: indoor residual spray
