# Supplementary figures and images for: Using sibship reconstructions to understand the relationship between larval habitat productivity and oviposition behaviour in Kenyan Anopheles arabiensis
Source: Malar J. 2019 Aug 23;18:286. doi: 10.1186/s12936-019-2917-5 (PMC6708163; doi:10.1186/s12936-019-2917-5)

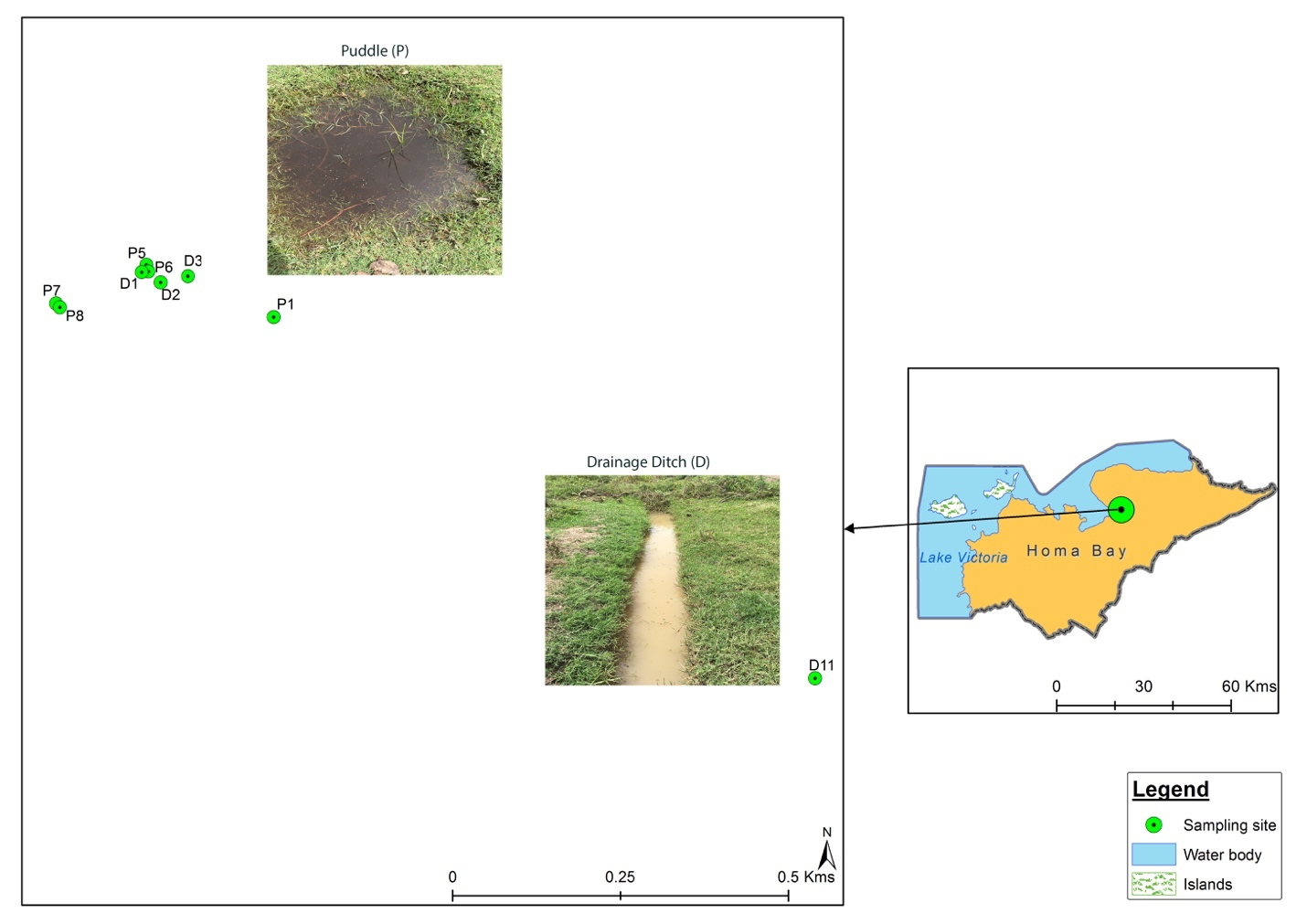

Supplement: Supplementary file 1 — Additional file 1. Schematic view of larval sampling sites at Oluch-Kimira rice irrigation scheme in Homa-Bay county, western Kenya. In the site labels, prefix P is puddle while D is drainage ditch. [file 12936_2019_2917_MOESM1_ESM.png]
