# Supplementary material for: Using sibship reconstructions to understand the relationship between larval habitat productivity and oviposition behaviour in Kenyan Anopheles arabiensis
Source: Malar J. 2019 Aug 23;18:286. doi: 10.1186/s12936-019-2917-5 (PMC6708163; doi:10.1186/s12936-019-2917-5)
Supplement: Supplementary file 2 — Additional file 2. Mendelian test for allele inheritance for parents and offspring in family A. The markers with p values in bold indicate consistency with Mendelian allele segregation rule. N/A represent markers that did not amplify, and n/a represent homozygous alleles whose p values could not be calculated. [file 12936_2019_2917_MOESM2_ESM.docx]

**Additional file 2**: Mendelian test for allele inheritance for parents and offspring in family A. The markers with p values in bold indicate consistency with Mendelian allele segregation rule. N/A represent markers that did not amplify, and n/a represent homozygous alleles whose p values could not be calculated.

|  |  | **Allele frequencies** | |  |
| --- | --- | --- | --- | --- |
| **Marker** | **Allele** | **Observed** | **Expected** | **P value** |
| CDC22 |  | N/A | N/A |  |
| CDC46 | 250/265 | 9 | 11.5 | **0.4714** |
|  | 260/265 | 10 | 11.5 |  |
|  | 265/265 | 16 | 11.5 |  |
|  | 250/260 | 11 | 11.5 |  |
| CDC675 | 174/176 | 24 | 11.5 | 7.00E-06 |
|  | 174/178 | 0 | 11.5 |  |
|  | 176/176 | 8 | 11.5 |  |
|  | 176/178 | 14 | 11.5 |  |
| 2RiS5 | 248/248 | 13 | 11.5 | **0.8222** |
|  | 248/256 | 21 | 23 |  |
|  | 256/256 | 12 | 11.5 |  |
| Ag2:79 | 173/173 | 46 | 46 | **n/a** |
| CDC18 | 130/130 | 11 | 11.5 | **0.8536** |
|  | 130/132 | 14 | 11.5 |  |
|  | 130/138 | 10 | 11.5 |  |
|  | 132/138 | 11 | 11.5 |  |
| CDC28C | 219/219 | 46 | 46 | **n/a** |
| CDC32 | 173/173 | 21 | 23 | **0.6127** |
| CDC34 | 164/182 | 6 | 11.5 | **0.0762** |
|  | 164/184 | 11 | 11.5 |  |
|  | 184/184 | 14 | 11.5 |  |
|  | 182/184 | 18 | 11.5 |  |
| CDC40B | 220/226 | 25 | 23 | **0.1442** |
|  | 220/220 | 15 | 11.5 |  |
|  | 226/226 | 6 | 11.5 |  |
| CDC44 | 121/123 | 10 | 23 | 9.09E-06 |
|  | 121/121 | 25 | 11.5 |  |
|  | 123/123 | 11 | 11.5 |  |
| Ag2:144 | 166/166 | 21 | 23 | **0.5553** |
|  | 156/166 | 25 | 23 |  |
| Ag2:46 | 136/136 | 21 | 23 | **0.5553** |
|  | 136/144 | 25 | 23 |  |
| Ag3:128 | 118/118 | 29 | 23 | **0.0768** |
|  | 104/118 | 17 | 23 |  |
| Ag3:249 | 113/113 | 21 | 23 | **0.5553** |
|  | 113/119 | 25 | 23 |  |
